# Reconstruction of the Permittivity of Ex Vivo Animal Tissues in the Frequency Range 1–20 GHz Using a Water-Based Dielectric Model

**DOI:** 10.3390/s24165338

**Published:** 2024-08-18

**Authors:** Flavia Liporace, Gianluca Ciarleglio, Maria Gabriella Santonicola, Marta Cavagnaro

**Affiliations:** 1 Department of Information Engineering, Electronics and Telecommunications, Sapienza University of Rome, 00184 Rome, Italy; flavia.liporace@uniroma1.it; 2 Department of Chemical Engineering Materials Environment, Sapienza University of Rome, 00161 Rome, Italy; gianluca.ciarleglio@uniroma1.it (G.C.); mariagabriella.santonicola@uniroma1.it (M.G.S.)

**Keywords:** dielectric properties, biological tissues, water content

## Abstract

Several medical techniques are based on the application of electromagnetic fields (EMFs) on the human body with therapeutic and/or diagnostic aims. The response of human tissues to the applied EMF is mediated by the tissues’ dielectric properties, which must therefore be characterized at the frequencies of the considered technique. Due to the heterogeneity and complexity of biological tissues, it is necessary to know their properties in vivo for the specific condition of interest. Traditional techniques for the dielectric characterization of biological tissues are invasive and, as such, not adoptable for this aim. Accordingly, alternative sensors and/or sensing methods are needed. Recently, a new wideband spectroscopy technique was proposed, based on quantities derived from the Magnetic Resonance (MRI) signal. Among these quantities, the water content was proposed to evaluate the dielectric properties at frequencies around a few GHz. This work verifies the possibility of deriving tissues’ dielectric properties in the frequency range of 1–20 GHz based on knowledge of the water content. The water content was retrieved through a dehydration procedure for five different ex vivo tissues. The achieved results were compared with references from the literature.

## 1. Introduction

Several medical techniques are based on the application of electromagnetic fields (EMFs) on the human body with therapeutic and/or diagnostic aims [1]. Microwave imaging is an example of a diagnostic application whose aim is to reconstruct an anatomical area of the patient using a system of transmitting and receiving antennas [2]. Thermal ablation is, in contrast, a therapeutic technique that adopts fields at microwave frequencies for the destruction of tumoral cells [3]. Implanted sensors can be cited as a monitoring application that works at frequencies between hundreds of MHz and some GHz [4].

In order to optimize EMF-based medical techniques, improving their efficacy and safety, an accurate reconstruction of the dielectric properties of the patient is needed. Biological tissues are heterogeneous and complex. Microscopically, cells and ions immersed in fluids are present; additionally, several cell types with different characteristics are found in the different tissues [5]. The tissue microscopic composition is fundamental to determining the biological effects of EMF at the level of cellular membranes or other structures. In this work, our interest is focused on medical applications of electromagnetic fields, such as oncological hyperthermia or thermal ablation. Accordingly, the average macroscopic values of dielectric properties are needed [6]. However, these properties change from subject to subject depending on different conditions [7]. For this reason, the dielectric characterization of the human body should be obtained specifically for the subject of interest. Traditional spectroscopy techniques that are adopted for the measurement of the dielectric properties of biological tissues are invasive and can only be applied on ex vivo tissues [8]. The open-ended probe technique, for example, is based on the use of a coaxial cable whose open end must be put in contact with the Material Under Test (MUT) [9]. Consequently, besides spectroscopy of the superficial skin layer, this procedure cannot be applied in vivo and does not represent a valid methodology for the specific dielectric characterization of patients. An alternative method is therefore needed.

Recently, the Magnetic Resonance (MRI) technique, commonly adopted for diagnostic imaging, was proposed for the derivation of the dielectric properties of in vivo subjects in a wide frequency band [10,11]. Indeed, it was found that, in the frequency range that goes from a few Hz to a few GHz, the dielectric properties of biological tissues depend on different factors [12]: at frequencies lower than 1 GHz, the main influence is the tissues’ histology and ionic currents, while at frequencies higher than 1 GHz, the main impact is from their water content [13]. Accordingly, in [10,11], a model for determining dielectric properties from data that were derivable from MRI was proposed. The model is based on the tissue’s water content at frequencies around a few GHz and on the so-called Electric Property Tomography (EPT) at lower frequencies. Joining the two pieces of information, dielectric properties on a wide frequency band were obtained. In [10,11], the model was applied using data obtained from the literature.

In this work, our attention is focused on the high-frequency part of the model, which depends on water content only [13] and which will be named, in the following, the water-based dielectric model.

Water is the principal component of biological tissues. Typically, tissues are divided into high-water-content (>50%) and low-water-content tissues (<50%). In the literature, different methods have been presented for the evaluation of the water content of tissues [14]. The dehydration technique, for example, is based on the heating of the tissues at high temperatures (50 °C or 100 °C) for a certain amount of time to induce the loss of water [15]. The needed amount of time depends on the chosen temperature and on the type of tissue that is considered. The water content is then derived through a weighting procedure while considering the relative percentage difference between the weight of the samples before and after the heating [15]. Through the freeze-drying procedure, in contrast, the water removal from the sample is obtained by bringing it to very low temperatures [16]. Also, in this case, the water content is determined through a weighting procedure. Both the dehydration and the freeze-drying procedures can be applied on ex vivo tissues, since manipulation of the samples is needed. The MRI technique can be applied instead on in vivo subjects to derive, through proper procedures, the spatial distribution of the water content in the tissues [17].

The aim of this work is to verify the hypothesis that at high frequencies, knowing the water content is sufficient to determine the dielectric properties of biological tissues [13]. The water content of ex vivo biological tissues (muscle, liver, heart, kidney and fat) was derived through the dehydration procedure. The knowledge of this quantity was then used to reconstruct the permittivity of the considered tissues in the frequency range of 1–20 GHz using the water-based dielectric model. The reconstructed values were compared, in the same frequency range, with data from the literature [18].

In the following, a theorical description of the dielectric behavior of biological tissues is provided first, and the structure of the water-based model is presented. Then, the dehydration procedure, adopted for the derivation of the water content, is described. Finally, the obtained results are presented and discussed.

## 2. Material and Methods

### 2.1. Dielectric Properties of Biological Tissues and Water-Based Dielectric Model

The dielectric behavior of biological tissues has been studied extensively in the literature, and many models have been proposed to describe it. The most adopted model, which is also considered in this work, is the Cole–Cole model [19]. It defines the dielectric behavior of biological tissues in the frequency range that goes from tens of Hz to tens of GHz with four dispersions mechanisms (α, β, γ, δ) [20]. Each dispersion involves a change in the dielectric properties with frequency and depends on specific biological characteristics [20]. The frequency range of interest in this work, i.e., the GHz region, is associated with the δ and γ dispersions, which are dependent on the relaxation of bounded and free water, respectively [20]. However, since the contribution of the δ dispersion on the dielectric behaviour of tissues is insignificant compared with the other ones, it can be neglected [21]. Consequently, in the GHz region, it is possible to only consider the presence of the γ dispersion and the water content of the tissues as the main influencing factors [20]. Considering these assumptions, the Cole–Cole model of the relative permittivity for one single dispersion (1-pole Cole–Cole model) can be represented in the following way:

(1) $$\varepsilon_{R}\left(\omega \right)={ɛ}_{\infty }+\frac{∆\varepsilon }{1+{\left(j\omega \tau \right)}^{\left(1-\alpha \right)}}+\frac{\sigma_{s}}{j\omega \varepsilon_{0}},$$

where ${ɛ}_{\infty }$ represents the permittivity at frequencies that are much higher than the relaxation one, while the term ${∆\varepsilon }_{\;}$ represents the difference between ${ɛ}_{s}$, the permittivity at frequencies that are much lower than the relaxation, and ${ɛ}_{\infty }.$ $\tau_{\;}$ in s indicates the relaxation time of the γ dispersion, and it is inversely related to the relaxation frequency ($\tau_{\;}=\frac{1}{{2\pi f}_{r}}$); *α* is a dispersion parameter whose values are included in the range of 0–1, and $\sigma_{s}$ S/m represents the static conductivity.

The Cole–Cole formula (Equation (1)) can be separated into the real ($\varepsilon_{R}^{\prime \;})$ and the imaginary ($\varepsilon_{R}^{\prime \prime \;})$ part, obtaining the following expressions:

(2) $$\varepsilon_{R}^{\prime }\left(\omega \right)=\varepsilon_{\infty }+\frac{\Delta \varepsilon \left[1+cos\left(\frac{\pi }{2}\left(1-\alpha \right)\right){\left(\omega \tau \right)}^{1-\alpha }\right]}{{\left[1+cos\left(\frac{\pi }{2}\left(1-\alpha \right)\right){\left(\omega \tau \right)}^{1-\alpha }\right]}^{2}+{sin}^{2}(\frac{\pi }{2}(1-\alpha )){\left(\omega \tau \right)}^{2(1-\alpha )}},$$

(3) $${\varepsilon^{\prime \prime }}_{R}\left(\omega \right)=\left(\Delta \varepsilon sin\left(\frac{\pi }{2}\left(1-\alpha \right)\right){\left(\omega \tau \right)}^{1-\alpha }+\frac{\sigma_{s}}{\omega \varepsilon_{0}}\right),$$

From Equations (2) and (3), it is evident how the static conductivity term ($\sigma_{s})$ is only present in the imaginary part of the permittivity.

The proposed water-based model simplifies the 1-pole Cole–Cole model (Equations (1)–(3)), neglecting the presence of the static conductivity term ($\sigma_{s}$) and fixing some parameters for all tissues and relating others to the tissues’ water content [10,11]. Accordingly, Equations (1)–(3) become the following equations:

(4) $$\varepsilon_{R}\left(\omega \right)={ɛ}_{\infty }+\frac{∆\varepsilon }{1+{\left(j\omega \tau \right)}^{\left(1-\alpha \right)}},$$

(5) $$\varepsilon_{R}^{\prime }\left(\omega \right)=\varepsilon_{\infty }+\frac{\Delta \varepsilon \left[1+cos\left(\frac{\pi }{2}\left(1-\alpha \right)\right){\left(\omega \tau \right)}^{1-\alpha }\right]}{{\left[1+cos\left(\frac{\pi }{2}\left(1-\alpha \right)\right){\left(\omega \tau \right)}^{1-\alpha }\right]}^{2}+{sin}^{2}(\frac{\pi }{2}(1-\alpha )){\left(\omega \tau \right)}^{2(1-\alpha )}},$$

(6) $$\varepsilon_{R}^{\prime \prime }\left(\omega \right)=\left(\Delta \varepsilon sin\left(\frac{\pi }{2}\left(1-\alpha \right)\right){\left(\omega \tau \right)}^{1-\alpha }\right),$$

With reference to the static conductivity, $\sigma_{s}$, this quantity depends on the extent of the ionic content and the ionic mobility of the biological tissues [21,22]. Since these quantities are influent in the low-frequency polarization mechanisms (α, β), $\sigma_{s}$ is not considered in the high-frequency water-based model (Equations (4)–(6)) adopted in this work.

The parameters ${ɛ}_{\infty },\;\alpha$ and τ were defined through an analysis of data from the literature [18]. In [18], the values of the dielectric properties are reported for each biological tissue and in the frequency range from 10 Hz to 40 GHz. Additionally, the corresponding parameters of the Cole–Cole model, obtained through a fitting procedure, are given. Focusing on the parameters of the Cole–Cole model for the γ dispersion, and comparing their values for the different tissues (muscle, liver, heart, kidney and fat), it was possible to assign fixed values to them [11]. In particular, the dispersion parameter α was fixed at 0.1 for all tissues, while the relaxation time τ was fixed to the one of free water (6.36 ps [23]). The parameter $\varepsilon_{\infty }$ was fixed at 4 and 2.5 for high-water-content tissues (>50%) and low-water-content ones (<50%), respectively [18].

The remaining parameter, i.e., $\varepsilon_{s}$, was obtained from the water content through the mixtures’ theory [14]. This theory is based on formulas that assume biological tissues as a mixture of water, which is the main phase, and biological structures that represent the solid inclusions. Through the use of these formulas, it is possible to determine the permittivity of a mixture based on knowing its water content. Among the many mixture formulas that are present in the literature, Fricke’s one was considered for high-water-content tissues, i.e., muscle, liver, heart and kidney [24]:

(7) $$\varepsilon_{s}=\varepsilon_{w}(\frac{1-P}{1+\left(K-1\right)P})(1+\frac{KP\varepsilon_{p}}{\varepsilon_{w}(1-P)})\;\mathrm{with}\;K=\frac{\left(1+x\right)}{x}+\frac{\varepsilon_{P}}{\varepsilon_{w}},$$

while Maxwell’s formula was used for the low-water-content tissues, i.e., fat [25].

(8) $$\varepsilon_{s}=\varepsilon_{w}\frac{2\varepsilon_{w}+\varepsilon_{p}-2P(\varepsilon_{w}-\varepsilon_{p})}{2\varepsilon_{w}+\varepsilon_{p}+2P\left(\varepsilon_{w}-\varepsilon_{p}\right)},$$

In Equations (7) and (8), P represents the volume fraction of the solid inclusions, and (1 − P) is the volume fraction of water. $\varepsilon_{w}$ is the relative permittivity of water at 100 MHz (equal to 78) and $\varepsilon_{p}$ the one of solid inclusions [23,26]. The value of $\varepsilon_{p}$ is 5 in Fricke’s formula (Equation (7)) [23] and 2.5 in Maxwell’s one (Equation (8)) [26]. The factor K depends on the permittivity of the inclusions and on their shape: x is 2 for spheres and 1.5 for prolate ellipsoids [23].

### 2.2. Sample Preparation and Water Content Evaluation

In this work, the dehydration method was adopted to determine the water content of muscle, liver, heart, kidney and fat. The tissues were purchased from a local slaughterhouse and stored at 4 °C until used in the experiment. Each tissue was cut into 4 or 5 cubic samples of approximately 2.5 cm × 2.5 cm × 3 cm.

After the samples were prepared, they were weighed and placed in Petri dishes with a diameter of 4 cm each and placed in the oven (Termostabil K_2_ oven). The heating at high temperatures caused evaporation and, thus, the loss of water that was present in the samples. This process took a different amount of time depending on the type of tissue. After 24 h of heating, the samples were taken out of the oven, and the weights of the samples were measured. In the case of the fat, the weights of the samples were measured for the second time after 48 h from the beginning of the procedure, while for the other tissues, a time interval of 96 h was applied. In fact, due to the low water content of fat, it was expected that the loss of water from the samples would require a shorter period compared to the other tissues with higher water contents. The samples were inserted again in the oven, and the procedure was repeated several times until the weight remained constant, i.e., the variation in the weight of the sample was lower than 0.5% between two successive measurements. When this latter condition was reached, the procedure was considered concluded, and the water was assumed to be completely lost by the tissue.

In the literature, commonly adopted temperatures for the dehydration of ex vivo biological tissues are 100 °C [23] and 50 °C [27]. In this work, a first session of dehydration was performed on muscle tissue to identify the suitable oven temperature. In particular, two dehydration sessions were conducted heating the muscle samples at 100 °C and 50 °C and comparing the results obtained in the two cases. As is shown in Appendix A, it was found that the use of a higher temperature provides the same results as the lower temperature but in a shorter amount of time. For this reason, the dehydration procedure was conducted on all other tissues at 100 °C.

The water content (wc) value was derived for each sample using the following formula [28]:

(9) $$wc=\frac{W_{w}-W_{d}}{W_{w}},$$

where $w_{w}$ in g is the weight of the wet sample (before dehydration), and $w_{d}$ in g is the weight of the dry sample (after the dehydration). Samples were weighted with a balance (Sartorius Entris II, BCE Model [29]) with a readability of 10 mg.

For each tissue type, the measured water content was presented on a mass basis with its average value and standard deviation. These values were computed following the procedure presented in Appendix B.

Since the adopted model depends on mixture formulas (Equations (7) and (8)), which are based on the water content on a volume basis, it was necessary to compute this quantity. To achieve that, the formula used above for the determination of the water content on a mass basis (Equation (9)) was rewritten for the volume case.

(10) $$wc_{volume}=\frac{V_{w}-V_{d}}{V_{w}}=\frac{V_{water}}{V_{w}},$$

where $V_{W}$ in m^3^ is the volume of the wet samples (before desiccation), $V_{d}$ in m^3^ is the volume of the dry samples (after desiccation), and $V_{water}$ in m^3^ is the volume of the water contained in the samples. The volume of the wet samples was measured before the heating procedure. Since the volume of water can be indirectly derived from its mass (m_water_ in g) and its density (ρ_water_ in kg/m^3^), Equation (10) was rewritten in the following way:

(11) $${wc}_{volume}=\frac{V_{water}}{V_{w}}=\frac{\frac{m_{water}}{\rho_{water}}}{V_{w}}\;,$$

where the density of water was 997 kg/m^3^ [30], and the mass of water was obtained from the difference between the weight of the wet samples and the weight of the dry ones. The uncertainty associated with the water content on a volume basis was computed as shown in Appendix B.

### 2.3. Calculation of Variability of Results

The dielectric properties that were reconstructed with the model depend on the water volume fraction of the tissues and, consequently, are affected by the uncertainty associated with this quantity (Appendix B). For this reason, it is necessary to evaluate the variability of the model’s results, generated by the uncertainty of the water volume fraction. In order to achieve that, the dielectric properties of the tissues were reconstructed using the average water volume fraction first and then the one associated with the uncertainty. The difference between the two cases was computed and considered to be the uncertainty-related variability of the results.

## 3. Results

In the following sections, the obtained results are presented. First, the water content values derived using the dehydration procedure are reported. Then, the tissues’ dielectric properties, reconstructed with the water-based model in the frequency range of 1–20 GHz, are shown and compared with values taken from the literature [18].

The scientific source taken as a reference [18] is a detailed database which collects the results obtained from the works of Gabriel et al. [20,21,22]. In these works, the dielectric properties of the main biological tissues were measured in the frequency range of 10–40 GHz. These measured data are widely used in the literature and represent, nowadays, the most reliable reference for the dielectric properties of tissues.

### 3.1. Water Content Evaluation: Dehydration Procedure

**Figure 1:**
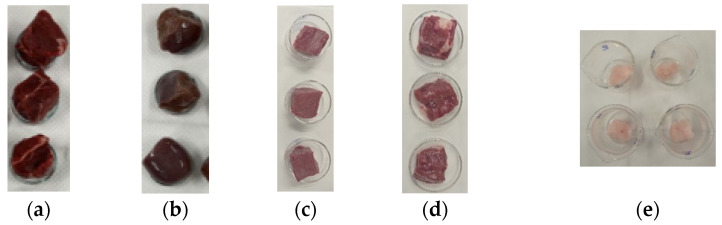
(**a**) Muscle; (**b**) liver; (**c**) heart; (**d**) kidney; and (**e**) fat samples before the dehydration procedure.

**Figure 2:**
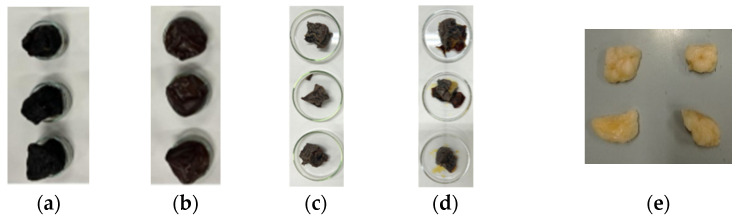
(**a**) Muscle; (**b**) liver; (**c**) heart; (**d**) kidney; and (**e**) fat samples after the dehydration procedure.

Figure 1 and Figure 2 show the samples before (Figure 1a–e) and after (Figure 2a–e) the dehydration procedure conducted using a temperature of 100 °C.

**Figure 3:**
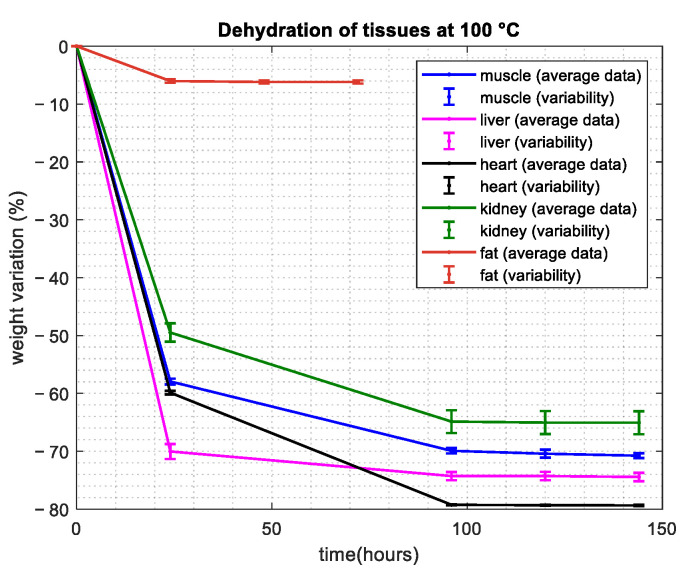
Temporal behavior of the percentage weight variation.

Depending on the tissue type, a different amount of time was needed in order to reach the end of the dehydration, i.e., the total loss of water. Figure 3 shows the percentage variation in the weight of the considered tissues with time. The average weight values and the standard deviation, computed on all samples for each tissue, are shown with dots and vertical lines, respectively.

After the end of the dehydration procedure, the water contents of the five tissues were determined. Table 1 reports the water content values, on a mass and on a volume basis, obtained for muscle, liver, heart, kidney and fat. For each tissue, the average value and the uncertainty are reported. References from the literature for the water content on a volume basis are also reported.

### 3.2. Dielectric Property Reconstruction

The water content values obtained from the dehydration procedure were used to determine the permittivity of the samples. Table 2 shows the parameters of the water-based dielectric model. As previously presented, for muscle, liver heart and kidney, which are classified as high-water-content tissues, the parameter $\varepsilon_{\infty }$ was set to 4 and Fricke’s mixtures formula (Equation (7)) was adopted to derive $\varepsilon_{S}$, with a value of the parameter x equal to 2, obtaining the values of 51.47, 46.99, 64.02 and 49.59 for muscle, liver, heart and kidney, respectively. In the case of fat, in contrast, i.e., a low-water-content tissue, the parameter $\varepsilon_{\infty }$ was fixed at 2.5, and $\varepsilon_{S}$ was obtained using Maxwell’s formula (Equation (8)), resulting in a value of 3.930. The relaxation time τ and the dispersion parameter α were the same for all tissues and were equal to the relaxation time of water (6.36 ps) and to 0.1, respectively.

**Figure 4:**
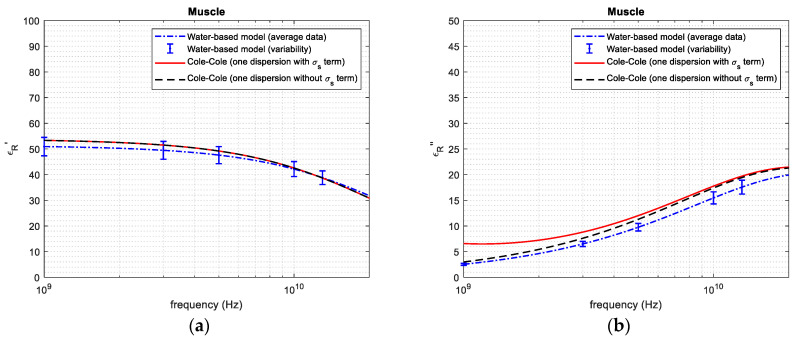
(**a**) Real and (**b**) imaginary part of permittivity of muscle.

**Figure 5:**
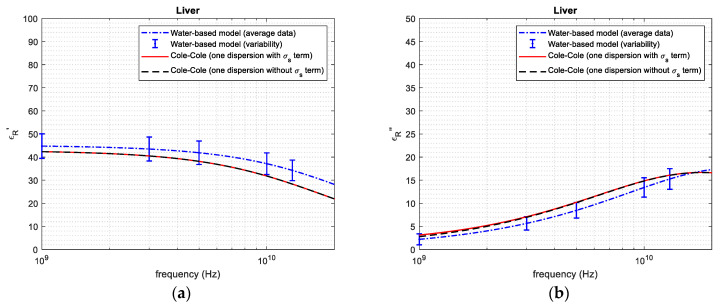
(**a**) Real and (**b**) imaginary part of permittivity of liver.

**Figure 6:**
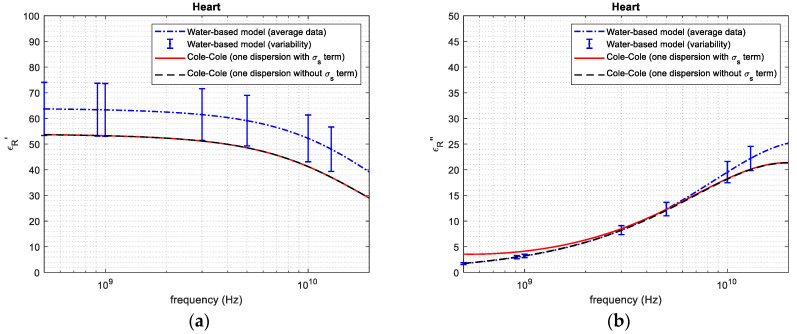
(**a**) Real and (**b**) imaginary part of permittivity of heart.

**Figure 7:**
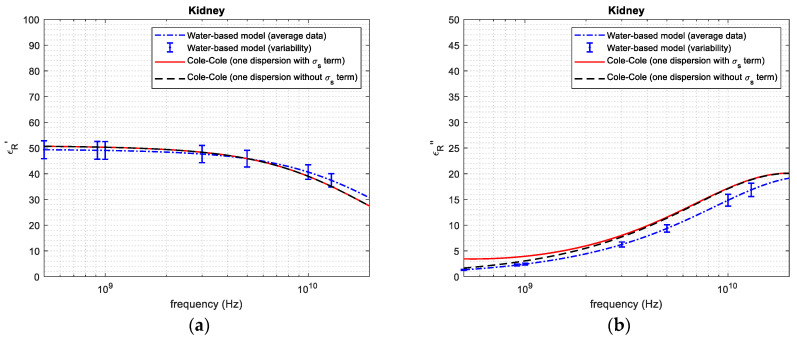
(**a**) Real and (**b**) imaginary part of permittivity of kidney.

**Figure 8:**
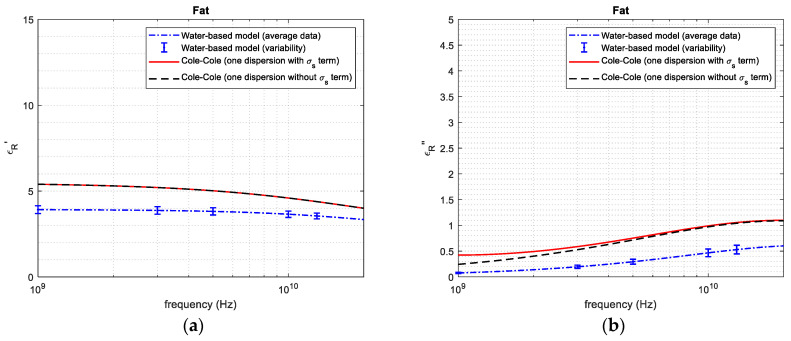
(**a**) Real and (**b**) imaginary part of permittivity of fat.

Figure 4, Figure 5, Figure 6, Figure 7 and Figure 8 report the (a) real and the (b) imaginary part of the permittivity of the five tissues in the frequency range of 1–20 GHz. The dashed–dotted blue curves represent the reconstruction obtained with the water-based model (Equations (4)–(6)) from the water content, derived through the dehydration procedure. The uncertainty of the data is represented with vertical bars. The continuous red curves and the dashed black ones represent the reference from [18], adopting the 1-pole Cole–Cole model (Equations (1)–(3)) with and without the term being dependent on the static conductivity, respectively.

Table 3 shows a comparison between the average results obtained with the water-based model and the Cole–Cole model [18] without the static conductivity term for the five tissues at five frequencies in the considered range (1 GHz, 3 GHz, 5 GHz, 10 GHz, 20 GHz). The values of the real and the imaginary parts of permittivity are reported, as well as the percentage difference.

## 4. Discussion

The results presented in this work show the possibility of reconstructing dielectric properties based only on knowing the water content.

In this work, the water contents of five different tissues were calculated through the dehydration procedure (Figure 3, Table 1). During the procedure, weighting sessions for the fat samples were repeated at intervals of 24, 48 and 72 h from the beginning of the procedure, with a constant weight being reached after 48 h. For the other tissues, in contrast, due to the higher water content compared with fat, longer time intervals were needed to achieve a constant weight of the samples. As is evident from Figure 3, for these tissues, the dehydration was considered to be completed after 120 h.

The obtained values of the water content on a volume basis show, for each tissue, higher variabilities with respect to those on a mass basis due to the high level of uncertainty in the measured samples’ volume (Table 1). For all tissues, considering the variabilities associated with the results, the water content obtained from the dehydration procedure agrees with the reference values.

Based on the water content, it was possible to apply the water-based model to calculate the permittivity of the different tissues (Figure 4, Figure 5, Figure 6, Figure 7 and Figure 8). From the figures, it is possible to note that—as derived from Equations (2) and (3)—the presence of the term that is dependent on the static conductivity only influences the imaginary part of permittivity. The behavior of the Cole–Cole model with (continuous red curve) and without (dashed black curve) the σ_s_ term is in fact the same for the real part of permittivity, while it shows some differences for the imaginary part, especially at frequencies lower than 2–3 GHz. It is also worth noticing that the presence of static conductivity influences the imaginary part of the permittivity of muscle, heart and fat, while its influence is negligible for liver and kidney. Since the water-based model does not consider the term that is dependent on the static conductivity, the imaginary part of permittivity obtained through the model follows the Cole–Cole reference without the σ_s_ term best. For the high-water-content tissues (Figure 4, Figure 5, Figure 6 and Figure 7), the results obtained with the water-based model, considering the associated variabilities, are in good agreement with the Cole–Cole models that were taken as references. For the low-water-content tissue, i.e., fat, in contrast, the water-based model gives a real and an imaginary part that are lower than those obtained from the reference. This can be explained, since fat is a heterogeneous tissue in which some blood infiltration could be present, as is also evidenced by the great variability in water content found in the literature (Table 1). Indeed, the average water content obtained for fat in this work corresponds to the minimum value of the range of values reported in the literature for this quantity (Table 1). In this respect, it is worth recalling that the literature data refer to different samples than those used in this work. Additionally, the reference dielectric properties are obtained from a work [18] that is different to that used to compare the water content of the tissue [14,31], and no information is given in [18] about the water content of the measured samples. Consequently, some differences between the obtained results and the reference are expected.

Comparing the dielectric properties calculated using the water-based model with those from the literature [18], at the frequencies reported in Table 3, the obtained maximum percentage difference in the real part of the permittivity is 5.435% for muscle, 27.71% for liver, 34.13% for heart, 11.94% for kidney and 27.5% for fat. Regarding the imaginary part, the maximum difference was found to be 13.35% for muscle, 18.24% for liver, 17.79% for heart, 20.29% for kidney and 69.3% for fat. The percentage difference varies depending on the tissue and on the frequency, reaching the highest values in the case of fat. It is worth recalling here that the comparison of the dielectric property values was performed between different tissue samples (those used in this work and those used in the used reference). Regarding the quite big difference obtained in the case of fat, in addition to the explanation given above, it must be noted that its dielectric properties are characterized by lower values with respect to those of the other tissues, leading, consequently, to high percentage differences.

In [31], the dielectric properties of ex vivo tissues were evaluated with a similar approach to the one proposed in this work. In particular, the water content was determined through the dehydration procedure, and mixture formulas were used. However, in [31], the mixture formulas were applied to obtain the dielectric permittivity of the tissues using as permittivity of the solid inclusion the values measured on the dried tissue. Accordingly, to apply the approach proposed in [31], additional measurements and related information besides the water content are needed. In this work, in contrast, the dielectric properties are derived based only on knowing the water content. This information, as previously reported, can be obtained from techniques such as, e.g., MRI, i.e., also in vivo.

Due to the procedure adopted for the derivation of the water content, the model was applied in this work on ex vivo tissues. In order to make the approach useful for medical techniques, it is necessary to verify its validity in the in vivo case too.

## 5. Conclusions

This work measured the water content of five ex vivo tissues and, starting from this information, adopted a water-based dielectric model for the reconstruction of the dielectric properties of biological tissues in the frequency range of 1–20 GHz. Ex vivo biological samples of muscle, liver, heart, kidney and fat were considered. Their water contents were derived using the dehydration procedure. The evaluated water contents were compared with literature data, finding an optimum agreement. Then, the dielectric properties evaluated with the water-based model were compared with values taken from the literature. A satisfactory agreement was obtained for all considered tissues, with the exception of fat. It is worth recalling here that the literature data do not reflect the specificity of the samples considered in this work, and that fat can show very different properties according to the blood infiltration level. To conclude, this work showed the suitability of deriving dielectric properties of biological tissues at a high frequency based only on knowledge of the water content. Since the water content can be obtained from the MRI signal, newly proposed techniques to derive tissues’ dielectric properties in vivo and specifically for each patient based on MRI can be developed.

## Figures and Tables

**Table 1:** Water content values of ex vivo tissues.

Tissue	Average and Standard Deviation—Mass Basis (%)	Average and Standard Deviation—Volume Basis (%)	Reference from the Literature—Volume Basis (%)
Muscle	70.8 ± 0.700	75.2 ± 5.22	73–78 [14]
Liver	74.4 ± 1.60	70.1 ± 5.47	73–77 [14]
Heart	79.3 ± 0.230	87.9 ± 6.52	86–87 [31]
Kidney	65.1 ± 3.99	73.1 ± 5.31	78–79 [14]
Fat	6.20 ± 0.620	5.20 ± 10.9	5–20 [14]

**Table 2:** Parameters of the water-based dielectric model for the considered tissues.

Tissue	$\varepsilon_{\infty }$	$\varepsilon_{s}$	τ (ps)	α
Muscle	4	51.47	6.36	0.1
Liver	4	46.99	6.36	0.1
Heart	4	64.02	6.36	0.1
Kidney	4	49.59	6.36	0.1
Fat	2.5	3.930	6.36	0.1

**Table 3:** Comparison between the results obtained with the model and reference [18] for muscle, liver, heart, kidney and fat tissues.

	Frequency	$\varepsilon_{R}^{\prime }$ (Water-Based Model)	$\varepsilon_{R}^{\prime }$ (Reference from the Literature [18])	${∆\varepsilon }_{R}^{\prime }$ (%)	$\varepsilon_{R}^{\prime \prime }$ (Water-Based Model)	$\varepsilon_{R}^{\prime \prime }$ (Reference from the Literature [18])	${∆\varepsilon }_{R}^{\prime \prime }$ (%)
	1 GHz	50.98	53.91	5.435	2.560	2.910	12.03
**Muscle tissue**	3 GHz	49.46	51.55	4.054	6.511	7.511	13.31
	5 GHz	47.60	49.23	3.311	9.791	11.30	13.35
	10 GHz	42.16	42.61	1.056	15.46	17.60	12.16
	20 GHz	31.86	30.86	3.240	19.78	21.41	7.613
	1 GHz	44.75	42.34	5.692	2.281	2.790	18.24
	3 GHz	43.48	40.49	7.384	5.650	6.771	16.55
**Liver tissue**	5 GHz	41.87	38.27	9.407	8.470	10.21	17.04
	10 GHz	37.14	31.74	17.01	13.43	14.81	9.318
	20 GHz	28.11	22.01	27.71	17.27	16.59	4.099
	1 GHz	63.33	53.25	18.93	3.210	3.251	1.261
	3 GHz	61.47	51.17	20.13	8.230	8.220	0.1216
**Heart tissue**	5 GHz	59.13	48.53	21.84	12.28	12.11	1.404
	10 GHz	52.24	41.02	27.35	19.54	18.21	7.304
	20 GHz	39.77	29.65	34.13	25.15	21.35	17.79
	1 GHz	49.06	50.30	2.465	2.431	3.050	20.29
	3 GHz	47.66	48.35	1.427	6.250	7.700	18.83
**Kidney tissue**	5 GHz	45.87	45.89	0.0438	9.370	11.44	18.09
	10 GHz	40.64	39.06	4.045	14.84	17.05	12.96
	20 GHz	31.01	27.70	11.94	19.04	20.06	5.085
	1 GHz	3.91	5.39	27.5	0.0760	0.250	69.6
	3 GHz	3.87	5.21	25.7	0.191	0.521	63.3
**Fat tissue**	5 GHz	3.82	5.00	23.6	0.290	0.721	59.8
	10 GHz	3.65	4.59	20.5	0.467	0.970	51.8
	20 GHz	3.35	4.01	16.5	0.601	1.092	44.9

**Figure A1:**
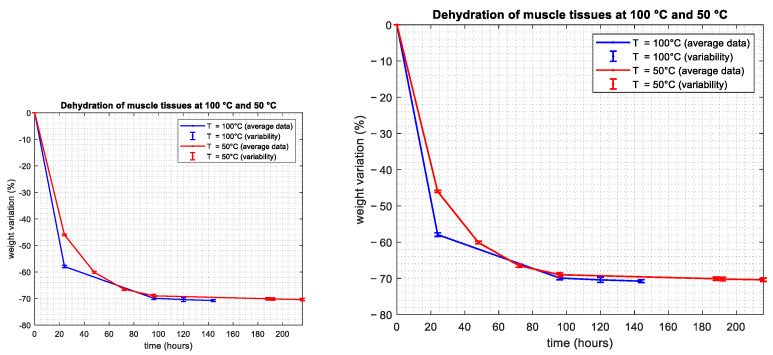
Temporal behavior of the percentage weight variation of muscle tissue heated at 100 °C and 50 °C.

## Data Availability

Data are contained within the article. Raw data available on request from the authors (F.L.).

## Appendix A

Two dehydration sessions were conducted on muscle samples using the two temperatures that are most often adopted in the literature for this procedure, i.e., 100 °C and 50 °C. Since the two temperatures were adopted in two independent experimental sessions, different muscle tissues were used and cut into five samples each. Figure A1 shows the percentage variation in the muscle tissue’s weight with time for the two different cases, representing, consequently, the temporal behavior of the water content loss. The blue and the red curves represent the average percentage variation in weight computed among all the samples of the muscle tissue when heated at 100 °C and at 50 °C, respectively. The vertical lines represent the variability.

From the figure, it is possible to notice a different temporal behavior of the dehydration procedure depending on the adopted temperature. As expected, dehydration is faster at 100 °C than at 50 °C. While in the first case, 120 h were needed to reach the equilibrium of the sample weight, in the second case, it was necessary to wait 192 h. The final weight loss was 70.8 ± 1.55% and 72.6 ± 3.37% for 100 °C and 50 °C, respectively. The slight difference in the final weight loss can be explained, considering that the muscle tissues adopted in the two sessions were different. It is worth noting that according to the literature, the percentage of water in the muscle tissue on a mass basis is 72 ± 0.7% [31], which is in agreement with the data obtained in this work. Through this analysis, it was found that using a higher temperature leads to the same result but in a shorter amount of time. For these reasons, in this work, a temperature of 100 °C was chosen to heat all other ex vivo tissues.

## Appendix B

For each tissue type, for the determination of the water content on a mass basis, the average value ($\left|x\right|$) and the standard deviation (sd) among the different samples were computed using the following formulas:

(A1) $$\left|x\right|=\frac{1}{N}\sum_{i=1}^{N}x_{i},$$

(A2) $$sd=\sqrt{\frac{\sum_{i=1}^{n}{\left(x_{i}-\left|x\right|\right)}^{2}}{N(N-1)},}$$

where $x_{i}$ is the i-th measurement and N is the number of samples.

The measured water content on a volume basis (Equation (10)) is associated with an uncertainty term that is dependent on two contributions. The first contribution is given by the variability of the mass of water that was measured through the weighting procedure. The second contribution is represented instead by the variability of the wet samples’ measured volumes.

With reference to the uncertainty contribution caused by the measurement of the water mass, this is, in turn, determined by two contributions: the precision of the mass measurements and the samples’ variability. The scale introduces two terms associated with the measurement accuracy, readout ($a_{read}$ = 0.01 g, [29]) and linearity ($a_{lin}$ = ±0.006 g [29]). Both accuracy components show a rectangular distribution, so that their contribution should be divided by $\sqrt{3}$ in order to obtain the corresponding uncertainty [32]. The uncertainty contribution related to the samples’ variability can be calculated as the standard deviation of the mean (SDM) among the values determined for the samples of each tissue. Therefore, the combined uncertainty of mass (δm) can be expressed as follows [32]:

(A3) $$\frac{\delta m}{m_{water}}=\sqrt{{\left(\frac{a_{read}}{m_{water}}\frac{1}{\sqrt{3}}\right)}^{2}+{\left(\frac{a_{lin}}{m_{water}}\frac{1}{\sqrt{3}}\right)}^{2}+{\left(\frac{SDM}{m_{water}}\right)}^{2}},$$

The samples’ volumes were measured using a ruler whose resolution is 0.1 cm, and the measurements were repeated three times for each edge of each sample. Accordingly, the uncertainty in the volume evaluation is determined by two factors: the one related to the shape of the sample, determined by the standard deviation of the mean (SDM) of the three repeated measurements, and the one related to the ruler accuracy, which shows a rectangular distribution and as such is obtained by dividing the rule’s accuracy by $\sqrt{3}$. Thus, the combined volume uncertainty (δV) is given by the following equation [32]:

(A4) $$\frac{\delta V}{V_{W}}=\sqrt{{\left(\frac{\delta l_{1}}{l}\right)}^{2}+{\left(\frac{\delta l_{2}}{l}\frac{1}{\sqrt{3}}\right)}^{2}+{\left(\frac{\delta w_{1}}{w}\right)}^{2}+{\left(\frac{\delta w_{2}}{w}\frac{1}{\sqrt{3}}\right)}^{2}+{\left(\frac{\delta h_{1}}{h}\right)}^{2}+{\left(\frac{\delta h_{2}}{h}\frac{1}{\sqrt{3}}\right)}^{2},}$$

where l, w and h represent the three cubes’ edges; $\delta l_{1}$, $\delta w_{1}$ and $\delta h_{1}$ are the SDM of the three repetitive measurements on the five samples; and $\delta l_{2}$, $\delta w_{2}$ and $\delta h_{2}$ are equal to 0.1 cm.

Finally, the combined uncertainty (u) in the water volume fraction is given by

(A5) $$u=\sqrt{{\left(\frac{\delta m}{m_{water}}\right)}^{2}+{\left(\frac{\delta V}{V_{W}}\right)}^{2}},$$

## References

[1] Mattsson M.O., Simkò M. (2019). Emerging medical applications based on non-ionizing electromagnetic fields from 0 Hz to 10 THz. Med. Devices.

[2] Wang Z., Lim E.G., Tang Y., Leach M. (2014). Medical Applications of Microwave Imaging. Sci. World J..

[3] Ahmed M., Brace C.L., Lee F.T., Goldberg S.N. (2011). Principles of and Advances in Percutaneous Ablation. Radiology.

[4] Malik N.A., Sant P., Ajmal T., Ur-Rehman M. (2021). Implantable Antennas for BioMedical Applications. IEEE J. Electromagn. RF Microw. Med. Biol..

[5] Heymsfield S.B., Wang Z., Baumgartner R.N., Ross R. (1997). Human Body Composition: Advances in Models and Methods. Annu. Rev. Nutr..

[6] Paulides M.M., Stauffer P.R., Neufeld E., Maccarini P.F., Kyriakou A., Canters R.A., Diederich C.J., Bakker J.F., Van Rhoon G.C. (2013). Simulation techniques in hyperthermia treatment planning. Int. J. Hyperthermia.

[7] Peyman A., Kos B., Djokić M., Trotovšek B., Limbaeck-Stokin C., Serša G., Miklavčič D. (2015). Variation in dielectric properties due to pathological changes in human liver. Bioelectromagnetics.

[8] Gregory A.P., Clarke R.N. (2007). Dielectric metrology with coaxial sensors. Meas. Sci. Technol..

[9] Stuchly M.A., Athey T.W., Samaras G.M., Taylor G.E. (1982). Measurement of Radio Frequency Permittivity of Biological Tissues with an Open-Ended Coaxial Line: Part II—Experimental Results. IEEE Trans. Microw. Theory Techn..

[10] Liporace F., Cavagnaro M. (2022). Development of MR-based procedures for the implementation of patient-specific dielectric models for clinical use. J. Mech. Med. Biol..

[11] Liporace F., Cavagnaro M. wideband model to evaluate the dielectric properties of biological tissues from magnetic resonance acquisitions. Proceedings of the 2023 17th European Conference on Antennas and Propagation (EuCAP).

[12] Sebek J., Albin N., Bortel R., Natarajan B., Prakash P. (2016). Sensitivity of microwave ablation models to tissue biophysical properties: A first step toward probabilistic modelling and treatment planning. Med. Phys..

[13] Foster K.R., Scheppes J.L. (1982). Dielectric Properties of Tumor and Normal Tissues at Radio through Microwave Frequencies. J. Microw. Power.

[14] Pethig R., Kell D.B. (1987). The passive electrical properties of biological systems: Their significance in physiology, biophysics and biotechnology. Phys. Med. Biol..

[15] Ciarleglio G., Russo T., Toto E., Santonicola M.G. (2024). Fabrication of Alginate/Ozoile Gel Microspheres by Electrospray Process. Gels.

[16] Sahin S., Karabey Y., Kaynak M.S., Hincal A.A. (2006). Potential use of freeze-drying technique for the estimation of tissue water content. Methods Find. Exp. Clin. Pharmacol..

[17] Fatouros P.P., Marmarou A. (1999). Use of magnetic resonance imaging for in vivo measurements of water content in human brain: Method and normal values. J. Neurosurg..

[18] Andreuccetti D., Fossi R., Petrucci C. An Internet Resource for the Calculation of the Dielectric Properties of Body Tissues in the Frequency Range 10–100 GHz. IFAC-CNR, Florence (Italy), 1997. Based on Data Published by Gabriel, C.; et al. in 1996. http://niremf.ifac.cnr.it/tissprop/.

[19] Cole K.S., Cole R.H. (1941). Dispersion and Absorption in Dielectrics I. Alternating Current Characteristics. J. Chem. Phys..

[20] Gabriel C., Gabriel S., Corthout E. (1996). The dielectric properties of biological tissues: I. Literature survey. Phys. Med. Biol..

[21] Gabriel S., Lau R.W., Gabriel C. (1996). The dielectric properties of biological tissues: II. Measurements in the frequency range 10 Hz to 20 GHz. Phys. Med. Biol..

[22] Gabriel S., Lau R.W., Gabriel C. (1996). The dielectric properties of biological tissues: III. Parametric models for the dielectric spectrum of tissues. Phys. Med. Biol..

[23] Schepps J.L., Foster K.R. (1980). The UHF and microwave dielectric properties of normal and tumor tissues: Variation in dielectric properties with tissue water content. Phys. Med. Biol..

[24] Fricke H. (1924). A mathematical treatment of the electric conductivity and capacity of disperse systems: I. The electric conductivity of a suspension of homogeneous spheroids. Phys. Rev..

[25] Farace P., Pontalti R., Cristoforetti L., Antolini R., Scarpa M. (1997). An automated method for mapping human tissue permittivities by MRI in hyperthermia treatment planning. Phys. Med. Biol..

[26] Smith S.R., Foster K.R. (1985). Dielectric properties of low-water-content tissues. Phys. Med. Biol..

[27] Reinoso R., Telfe B.A., Rowland M. (1997). Tissue water content in rats measured by desiccation. J. Pharmacol. Toxicol. Methods.

[28] Liporace F., Ciarleglio G., Santonicola M.G., Cavagnaro M. Dielectric Characterization of Biological Tissues at Microwave Frequencies Based on Water Content. Proceedings of the 2024 18th European Conference on Antennas and Propagation (EuCAP).

[29] (2020). Entris II Advanced Line Operating Instructions.

[30] Lemmon E.W., Bell I.H., Huber M.L., McLinden M.O. (2018). NIST Standard Reference Database 23: Reference Fluid Thermodynamic and Transport Properties-REFPROP.

[31] Mohammed B.M., Mohamed M., Naqvi S.A.R., Bialkowski K.S., Mills P.C., Abbosh A.M. (2020). Using Dielectric Properties of Organ Matter and Water Content to Characterize Tissues at Different Health and Age Conditions. IEEE J. Electromagn. RF Microw. Med. Biol..

[32] Taylor B.N., Kuyatt C.E. (1994). Guidelines for Evaluating and Expressing the Uncertainty of NIST Measurement Results.

